# Mapping Emotional Pathways to Social Identity in Hybrid Work: A Computational Model for Organizational Cohesion

**DOI:** 10.3390/bs16020303

**Published:** 2026-02-21

**Authors:** Shuang Li, Jiajia Hao, Yining Chai, Tongyue Feng, Yuxin Liu, Xiaoxia Zhu

**Affiliations:** 1 Economics Management School, Yanshan University, Qinhuangdao 066000, China; lishuang820907@stumail.ysu.edu.cn (S.L.); chaiyining@stumail.ysu.edu.cn (Y.C.);; 2 Institute for Advanced Studies in Humanities and Social Sciences, Beijing Normal University, Zhuhai 519000, China; 202422109067@mail.bnu.edu.cn

**Keywords:** emotional contagion, social identity, organizational culture, computational modeling, hybrid work, micro-foundations

## Abstract

This study develops an integrated computational model to illuminate the micro-dynamics through which transient emotional contagion evolves into stable social identity within organizations, with a specific focus on hybrid work environments. Drawing on organizational psychology and employing an agent-based modeling approach, we formalize a four-stage process—Emotional Cycle, Emotional Memory Accumulation, Cognitive Formation, and Enhancement Effect—that captures how fleeting affective experiences coalesce into enduring group identification. Our simulations reveal that cognitive heterogeneity moderates this pathway, leading to slower but more robust identity formation. Gender differences emerge as significant, with females demonstrating higher susceptibility to emotional contagion, while males’ identification is more strongly influenced by issue relevance. Crucially, exploratory simulations contrasting high- and low-hybridity configurations demonstrate that dispersed, digitally mediated work attenuates the emotional feedback loop, slows consensus formation, and heightens the risk of sub-group silos, thereby fundamentally reshaping the identity formation pathway. This research provides a mechanistic explanation of the emotional foundations of organizational culture and offers managers an evidence-based, dynamic framework for strategically cultivating collective identity in an increasingly hybrid world.

## 1. Introduction

In an era defined by digital interconnectedness and the rapid normalization of hybrid and remote work arrangements, the dynamics of emotion and identity have emerged as central, yet increasingly complex, themes in work and organizational psychology. While traditional research has provided robust insights into individual-level cognitive processes—perception, memory, decision-making—there is growing recognition that the most consequential organizational phenomena arise from the interactions between individuals (Forgas, 1995). From teams and departments to broader organizational cultures, these collective structures are not merely aggregates of individual attributes but emerge from the dynamic, often nonlinear, interplay of thoughts, feelings, and behaviors among members (Christov-Moore et al., 2014). This shift in perspective calls for a deeper investigation into the micro-foundations of macro-level organizational constructs, particularly within the evolving context of hybrid work, where the pathways linking transient, individual emotional experiences to stable, shared identities are being fundamentally reshaped.

Social identity theory has long offered a foundational framework for understanding how individuals derive self-concept from group membership and how this identification shapes behavior (Coviello et al., 2014). Yet, the precise mechanisms through which fleeting affective experiences—such as those propagated through emotional contagion—coalesce into enduring cognitive and social structures remain less clear. This gap is particularly salient and urgent in today’s hybrid and digitally mediated work environments. Here, emotional cues are rapidly transmitted across a mosaic of formal and informal networks, blending face-to-face exchanges with computer-mediated communication. The attenuation of non-verbal cues, the prevalence of asynchronous interactions, and the spatial fragmentation of teams potentially disrupt the traditional, proximity-based rhythms of emotional exchange and collective sense-making. Consequently, the pathway from micro-level emotional arousal to macro-level identity consensus in these settings is not only undertheorized but represents a critical puzzle for building cohesive organizational cultures.

Recent advances in organizational scholarship have begun to address this complexity by examining the microstructural and dynamic aspects of collective behavior (Dong et al., 2023). Building on work in this journal, researchers have emphasized the need to understand the dynamic interplay between affective processes and identity formation. Computational modeling and process theorizing are increasingly employed to capture the real-time interplay of cognition and emotion in groups, aligning with a broader movement toward studying processes rather than outcomes, and mechanisms rather than correlations (Miller & Page, 2007). These approaches are exceptionally well-suited to dissect the novel contingencies introduced by hybrid work, as they allow for the systematic manipulation of interaction patterns, communication delays, and network structures that define the hybrid experience (Assmann & Czaplicka, 1995).

This study contributes to this movement by proposing and testing an integrated computational model that elucidates how emotional contagion serves as an affective engine for social identity formation, with explicit relevance to the challenges of hybrid work. We ask: How do the fundamental processes of emotional contagion and identity formation translate to environments where social interactions are partially digitized and spatially dispersed? Drawing on theories of emotional contagion, social identity, and collective memory, we introduce a “Four-Stage, Four-Mechanism” framework that formalizes the transition from individual emotion to group-level identity. To examine these mechanisms, we employ an agent-based model simulating how emotional states diffuse through a network of heterogeneous agents and—under specific conditions—stabilize as social identity. This method allows us to explore the roles of cognitive heterogeneity, gender, emotional intensity, and attention, and to theoretically model how variations in interaction frequency and strength—hallmarks of hybrid work—might shape identity outcomes.

Our findings offer core contributions to work and organizational psychology, with specific implications for hybrid work. First, we provide a mechanistic explanation of how micro-level affective processes feed macro-level cultural stability. Second, we highlight the moderating role of cognitive heterogeneity, a finding that informs strategies for integrating diverse and often geographically dispersed talent. Third, we introduce temporal depth by conceptualizing identity as a diachronic process shaped by accumulated emotional memory—a crucial perspective for environments where shared physical experiences are less frequent. By situating emotion at the heart of identity formation and leveraging computational modeling to unpack its dynamics, this study bridges micro–macro divides and offers practitioners a refined, evidence-based toolkit for cultivating intentional and resilient organizational cultures in an increasingly hybrid world. Compared with existing research in organizational identity, this study introduces three moderating variables—cognitive heterogeneity, attentional allocation, and gender differences—into the model of group identity formation. It reveals how individual and group-level differences shape the transmission, interpretation, and internalization of emotional signals, thereby making the theoretical framework more aligned with the complexity and situational dependency of organizational realities.

## 2. Literature Review

### 2.1. Social Identity and Its Organizational Instantiation

Social Identity process, involves social categorization, social comparison, and the pursuit of positive distinctiveness, is a powerful force in organizational life (Ashforth & Mael, 1989). In organizations, social identity manifests as organizational identification—the extent to which employees define themselves by their affiliation, internalize its values, and experience its successes and failures as their own (van Knippenberg, 2020).

The construction of this identity is fundamentally cognitive (Barsade, 2002). Contemporary models posit that it forms through dual pathways: self-stereotyping, where individuals infer their own attributes from the group prototype, and self-anchoring, where they project their own attributes onto the group (Wagoner & Hogg, 2018). Recent work extends this understanding by emphasizing how organizational identity is dynamically reconstructed through leadership and shared storytelling (Haslam et al., 2020). A prerequisite for these processes is an individual’s cognitive and emotional appraisal of the group’s values and behaviors. In essence, the journey of social identity formation is a search for a sense of belonging and purpose, which translates to finding one’s place and meaning within the corporate culture (Wei et al., 2023).

### 2.2. Emotional Contagion as the Affective Underpinning of Culture

While identity is cognitively anchored, its formation is profoundly affective. Emotional contagion—the automatic tendency to mimic and synchronize emotional expressions with others, leading to emotional convergence—is a basic building block of social interaction (Lu & Hong, 2022). In organizations, this active driver of group affective tone and collective mood (Ni et al., 2020) creates what can be termed an “emotional cycle”: a loop of transmission → contagion → feedback → intensification among members. This cycle promotes homogenized emotional states and shared social cognition, fostering a sense of belonging to a group that “feels the same way,” thereby initiating social identification (Hatch & Schultz, 2000).

### 2.3. Integrating Emotion and Identity: Bridging the Micro–Macro Divide

Despite broad acknowledgment that emotion is a powerful driver of social identity, a significant gap remains in understanding the specific mechanisms that translate momentary, micro-level emotional contagion into stable, macro-level organizational identity. As Ellemers notes, while we understand the importance of moral emotions and group-based appraisals, the precise computational pathways through which transient emotions become stable identifications remain largely unexplored (Ellemers, 2021). Existing studies often adopt a homogenized, macro-level perspective, with limited consideration for the heterogeneous characteristics of individuals and the dynamic contexts they inhabit. Key questions about the precise pathways from fleeting affect to enduring identification, especially within complex modern organizational structures, are thus left inadequately explored.

### 2.4. The Hybrid Work Context: New Frontiers for Emotion and Identity Research

The rapid ascendancy of hybrid work models—characterized by the flexible blending of in-person and remote work, mediated by digital tools—introduces a novel and critical context that reconfigures the very terrain on which emotion and identity interact (Zhao & Xue, 2023). This environment is defined by reduced spontaneous co-location, a heavier reliance on computer-mediated communication (CMC), and the interweaving of synchronous and asynchronous interactions (Liu & Hilton, 2005).

Emerging research has begun to chart the implications of this shift. Studies on emotional contagion suggest that while digital channels can transmit affect, the process is often less efficient and more prone to ambiguity (Zhang & Shi, 2022). Moreover, the physical asynchrony of remote work carries the risk of work-related isolation, potentially undermining employees’ collective identity (Ajzen & Taskin, 2021). Virtual communication platforms often lack in-depth dialogue and exhibit weaker emotional interaction, which can also negatively impact the sense of belonging among expatriate personnel (Bianchi et al., 2023; Mumtaz, 2024). The richness of emotional cues is filtered through video screens and text, potentially dampening the intensity of contagion or leading to misinterpretations. Conversely, the permanence and visibility of digital emotional expressions (e.g., in chat logs) might create new forms of affective resonance (Stieglitz & Dang-Xuan, 2013). Research on social identity in virtual teams highlights the role of digital symbols, shared virtual spaces, and concerted communication rituals in fostering a sense of “virtuality” and team identification. Furthermore, the challenge of maintaining a cohesive organizational culture without constant physical presence has spurred interest in how cultural values and narratives are translated and sustained through digital means.

However, a notable limitation persists. Much of the existing literature on hybrid and remote work remains descriptive, correlational, or static, often documenting outcomes rather than illuminating the dynamic processes that lead to them (Barsade & Gibson, 2007). We lack a fine-grained, mechanistic understanding of how the altered interaction patterns, communication latencies, and modified social cues inherent to hybrid work influence the real-time, iterative processes of emotional contagion and the subsequent crystallization of identity. There is a pressing need for theoretical frameworks and methodologies capable of modeling these dynamic, non-linear pathways under the specific contingencies of hybrid work.

This study directly addresses this gap. The computational model we present is uniquely positioned to theorize and simulate these dynamic formation mechanisms within a hybrid context. By conceptualizing organizational networks and allowing parameters such as tie strength (simulating interaction frequency and media richness), attention (simulating the salience of digital versus physical cues), and feedback loops to be varied, our framework can model how the attenuated or altered social exchanges of hybrid work impact the journey from emotional stimulus to stable group identification. It thus moves beyond static descriptions to offer a generative, process-oriented perspective on building and sustaining organizational identity in an increasingly hybrid world.

### 2.5. Theoretical Synthesis and Research Position

In summary, while social identity theory and emotional contagion research provide a solid foundation, and while nascent work on hybrid contexts identifies key challenges, a crucial theoretical and methodological integration is missing. The present research positions itself at this intersection. We synthesize these streams to propose a dynamic, multi-stage model that specifies the mechanisms linking emotion to identity. Crucially, we employ an agent-based computational methodology that not only formalizes this theory but also provides a versatile platform for explicitly simulating and examining how these mechanisms operate under the distinct conditions—such as altered network structures and communication dynamics—that define hybrid work environments. This approach allows us to open the “black box” of identity formation with a precision particularly suited to the complexities of the contemporary workplace.

## 3. Theoretical Framework: An Integrated Model of Emotion and Identity Formation in Organizations

**Figure 1:**
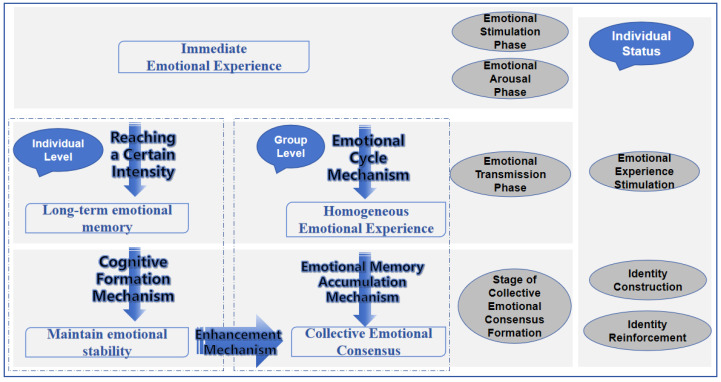
The Integrated “Four-Stage, Four-Mechanism” Model from Emotional Contagion to Social Identity.

Against the backdrop of the evolving hybrid work landscape, we integrate the mechanisms from emotional triggering to identity formation and enhancement within organizations into a coherent framework, drawing upon the theoretical foundations of emotional contagion theory, social identity theory, and organizational memory theory. This framework traces the pathway from transient affective experiences to stable collective identity, conceptualized through four evolutionary stages, driven by four core mechanisms, as illustrated in Figure 1.

The Four Evolutionary Stages are:

1. Emotional Stimulus: An individual encounters emotion-laden information through social or organizational channels;
2. Emotional Arousal: The stimulus triggers an immediate affective response;
3. Emotional Dissemination: The aroused emotion motivates the individual to share and diffuse this feeling within their interpersonal network;
4. Formation of Group Emotional Consensus: Individual emotions converge, forming a shared affective tone that serves as the foundation for social identity.

These stages are propelled by the following interconnected mechanisms. In Section 4.4, “Model Dynamics and Mechanism Implementation”, the emotional warmth mechanism, emotional memory accumulation mechanism, cognitive formation mechanism, and enhancement mechanism are simulated by setting dynamic rules for the state of cells.

### 3.1. The Emotional Cycle Mechanism: The Engine of Affective Convergence

Emotional contagion is a foundational mechanism of social cohesion. We conceptualize this as an Emotional Cycle, where the expression of emotion elicits feedback (e.g., resonance, agreement) from others, which in turn intensifies the original expresser’s own emotional state. This “expression-feedback-intensification” loop is crucial for understanding how isolated feelings amplify into group-level phenomena. In tightly knit teams, these cycles create a self-reinforcing system of affective exchange, forging initial group solidarity through shared affective experiences.

In hybrid environments, the synchronous, rich, and multi-modal “expression-feedback” loop of face-to-face interaction is often disrupted. Digital feedback (e.g., an emoji reaction in chat, a delayed text reply) is typically weaker, less immediate, and carries fewer nonverbal cues. This attenuation likely dampens the feedback factor, potentially slowing the intensification process and making it harder to achieve strong emotional convergence. Our computational model allows us to simulate this by adjusting the feedback parameter (fb) and the strength of network ties, enabling exploration of how different communication media—mimicking the blend of in-person and digital interactions—impact the efficiency of the emotional cycle. This provides a theoretical lens to examine why building collective enthusiasm or managing collective anxiety might require more deliberate effort in hybrid settings.

### 3.2. The Emotional Memory Accumulation Mechanism: The Sedimentation of Affective Culture

The Emotional Memory Accumulation mechanism posits that the homogenized affective states formed within emotional cycles are stored as latent affective resources. These “emotional memories” become part of an individual’s and a group’s affective repository, constituting the emotional undercurrent of culture. When similar situations recur, these accumulated memories are activated, filtering interpretations and guiding reactions. Collectively, they form the affective bedrock of an organization, allowing transient moods to sediment into enduring emotional dispositions.

Hybrid work modes can lead to a reduction in shared, high-intensity, co-located “key moments” (e.g., spontaneous celebrations, palpable tension in a meeting room). The affective landscape may become more fragmented, composed of frequent but lower-intensity digital interactions. This raises a critical question: Can a series of dispersed, digitally mediated emotional exchanges sediment into a coherent and potent “emotional memory” with the same identity-forming power? Our model suggests that the attention (*d*) parameter becomes even more critical in this context. For a digital interaction to contribute meaningfully to emotional memory, it must cut through the noise and be deemed personally relevant and salient. This underscores the importance for leaders and organizations to design intentionally salient digital interactions and rituals that can capture collective attention and serve as anchors for shared emotional memory in a hybrid world.

### 3.3. The Cognitive Formation Mechanism: From Affective Experience to Self-Concept

The establishment of a durable social identity requires conscious cognitive processing. The Cognitive Formation mechanism emphasizes the critical transition from “how I feel” to “what I think.” Here, aroused emotions and their accumulated memories are integrated into the self-concept through individual reflection and appraisal. An individual’s cognitive level—their tendency and capacity for deep, reflective thought—plays a critical moderating role, determining the length and complexity of this sense-making pathway.

Hybrid work, with its greater reliance on asynchronous, text-based communication (e.g., emails, project management tool comments), may inadvertently create more space for individual cognitive reflection. The constant, real-time social pressure of a physical office is relaxed, potentially elongating the cognitive appraisal process. In our model, this can be conceptualized as an effective increase in the cognitive pathway parameter (cognition) for many interactions. This has a dual-edged implication: it may slow down the speed of identity formation as individuals take longer to process, but it could also lead to a more robust and deliberate form of identification, as conclusions are reached through less impulsive, more reflective thought. This highlights a potential hidden strength of hybrid work: fostering a culture of more deliberate identification.

### 3.4. The Enhancement Effect Mechanism: Solidifying the Boundaries of “We”

Once an individual begins to identify with a group, the Enhancement Effect described by Social Identity Theory becomes pivotal. Driven by the motivation to maintain self-esteem, individuals cognitively exaggerate intra-group similarities and superiorities while accentuating differences with out-groups. This mechanism continually reinforces the symbolic boundaries of organizational identity at cognitive, affective, and behavioral levels.

In hybrid environments, “in-group” interaction often migrates to digital enclaves—dedicated team channels, private groups, or algorithmically curated feeds. This can powerfully amplify the enhancement effect, creating digital “echo chambers.” Within these closed loops, shared views are constantly reinforced with minimal exposure to dissenting perspectives, and group symbols are frequently circulated. Our model captures this dynamic through the enhancement coefficient (rise%). The hybrid context suggests this coefficient may be particularly high within digital in-groups, leading to rapid and potent solidification of sub-group identities. For organizational leaders, this presents both a challenge (fostering silos) and an opportunity: consciously designing digital spaces and communication flows that promote constructive cross-group exposure is essential to harness the enhancement effect for the broader organizational identity, rather than allowing it to fuel fragmentation.

### 3.5. Synthesis: A Dynamic System of Identity Emergence in Context

In summary, these four mechanisms constitute a coherent dynamic system that explains the micro-foundations of organizational culture. Crucially, this framework is not static but context-sensitive. As we have delineated, the transition to hybrid work environments systematically alters the operating conditions for each mechanism—moderating the efficiency of the Emotional Cycle, challenging the sources of Emotional Memory, extending the Cognitive Formation pathway, and potentially intensifying the Enhancement Effect within digital sub-networks. Our integrated model thus provides a granular, process-based lens not only for understanding identity formation in general but for diagnosing and intervening in the specific cultural dynamics of hybrid organizations. It allows us to move from asking if identity forms in hybrid work to theorizing how the process differs and what levers can be used to guide it towards positive outcomes.

## 4. Methodology: A Computational Model of Organizational Identity Formation

To investigate the dynamic pathway from emotional contagion to social identity, with particular relevance to the structures of hybrid work, we employ an agent-based computational model using a Cellular Automata (CA) framework. This approach is adept at capturing the emergent, bottom-up processes through which micro-level interactions give rise to macro-level organizational phenomena. By simulating how individual affective states evolve through social networks into stable collective identities, we can formalize and test our theoretical framework and, importantly, explore its sensitivity to the interaction patterns characteristic of hybrid environments.

This study examines how transient emotional experiences of individuals coalesce into stable collective emotional consensus, with emotional contagion mechanisms playing a pivotal role in this process. Cellular automata (CA) can demonstrate how interactions at the micro level influence individual states, ultimately giving rise to macro-level organizational phenomena. A cell’s next-step state is determined by its current state and the states of its neighbors, reflecting how a cell’s state is “infected” by its neighbors. This characteristic of cells automatically updating their states based on neighboring influences closely parallels how individuals experience emotional contagion within social networks, where others’ emotions trigger their own. Therefore, this paper employs cellular automata (CA) as a simulation tool. An individual cell’s emotional state changes after being infected by neighboring cells’ emotions. Through an emotional circulation mechanism and an affective memory accumulation mechanism, this emotional intensity continuously strengthens. Subsequently, via a cognitive formation mechanism, transient emotional experiences transform into stable affective attitudes. Ultimately, individuals shift their identity recognition, perceiving themselves as belonging to the ingroup. The initial transient emotional experience also evolves into stable group consensus.

The SIR model classifies individuals in infectious disease transmission into three states: S (susceptible), I (infected), and R (recovered). These three states align with individual states in emotional contagion. Therefore, the SIR model is employed to represent the state transitions of individuals from emotional contagion to social identification.

### 4.1. Model Structure and Organizational Correspondence

#### 4.1.1. Cellular Space and Organizational Context

**Figure 2:**
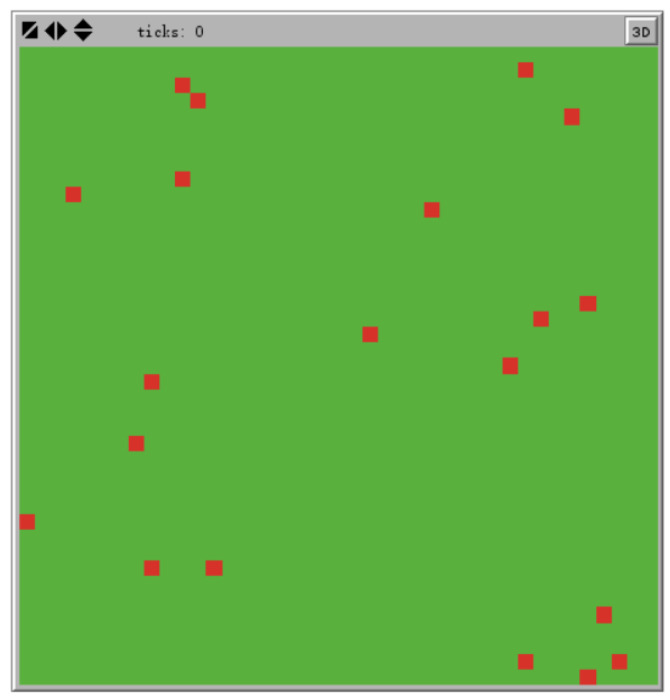
NetLogo World.

**Figure 3:**
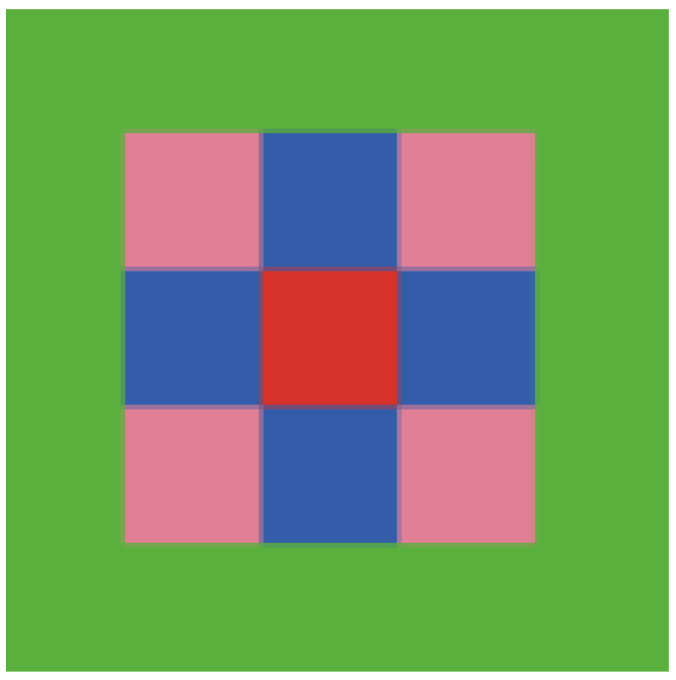
Moore-type Neighbors.

The model represents an organizational social network as a two-dimensional grid of 41×41 cells (total *N* = 1681), where each cell represents an organizational member. The grid employs periodic boundary conditions, creating a continuous interaction space. This structure is a generalized abstraction that can be mapped onto various organizational contexts, from co-located departments to the dispersed networks inherent in hybrid work arrangements, as illustrated in Figure 2 and Figure 3.

#### 4.1.2. Neighborhood Structure, Organizational Relationships, and Hybrid Work Mapping

We adopt a Moore neighborhood structure, where each agent’s state is influenced by its eight surrounding neighbors (Figure 3). Crucially, we distinguish between two network layers to reflect varying relationship strength:

Primary Network (vertical/horizontal neighbors): This represents an individual’s immediate team, close colleagues, or strong ties. In a hybrid context, these are relationships characterized by high-frequency interaction, which may occur through a blend of in-person and digital channels (e.g., daily stand-ups, close collaboration).

Secondary Network (diagonal neighbors): This represents weaker connections, such as cross-departmental contacts or infrequent interactions. In hybrid work, these ties are likely maintained almost exclusively through digital tools (e.g., enterprise social networks, email) with minimal to no physical co-presence.

This distinction is pivotal for modeling hybrid work dynamics. The relative density and influence strength of the Primary vs. Secondary network can be adjusted to simulate different hybrid configurations. For instance, a “sparser” Primary network (simulating fewer strong, frequent ties due to reduced office presence) directly models the attenuation of close physical contact, allowing us to test its impact on the emotional cycle. The model’s structure thus offers a flexible representation of the blended interaction ecology of hybrid organizations.

Given that interpersonal relationships within real-world organizations are often more complex, we now explore the impact of alternative network structures on the model. In scale-free and multi-network environments, connections between individuals are more extensive, particularly in cross-departmental collaboration scenarios where individuals receive emotional information from a greater variety of sources. Furthermore, connections may exhibit asymmetry, meaning emotional contagion between two nodes could yield differing effects based on the roles of the transmitter and receiver. For nodes with numerous neighbors—such as organizational leaders—the emotional information transmitted and feedback received from different neighbors will exert heterogeneous influences. Given that interpersonal relationships in real-world work environments are relatively fixed due to individual functional constraints, significant changes in network edges are unlikely to occur in the short term. Therefore, the impact of network dynamics on the model is not further discussed here.

It is worth noting that in scale-free networks, link connections exhibit heterogeneity, leading to the emergence of a small number of opinion leaders with many neighbors within the group. Given the greater influence of opinion leaders in the network, the extent to which individuals are affected by others’ emotional information during emotional contagion depends not only on the closeness of their interpersonal relationship with the emotional transmitter. For individuals connected to opinion leaders, the emotional information transmitted by these leaders may significantly amplify the emotional intensity of the receiver. For opinion leaders themselves, the emotional feedback loop mechanism ensures that they continuously receive feedback through ongoing emotional exchanges, further amplifying their own emotional intensity. Consequently, within scale-free networks, heightened individual emotional intensity may foster broader and tighter identity structures. However, due to the fragility inherent in scale-free networks, shifts in the emotional attitudes of individual opinion leaders toward the organization can induce significant volatility within the group.

#### 4.1.3. Agent States and Psychological Transitions

Agents transition through states corresponding to psychological positions in the identity formation process (see Table 1): Susceptible (S) individuals who can be influenced; Infected (I) individuals experiencing and disseminating active emotional arousal; and Recovered (R) individuals who have developed immunity, either through emotional decay (R1) or cognitive rejection (R2). These states map onto our theoretical framework of emotional arousal, cognitive processing, and identity formation.

### 4.2. Model Parameters and Their Theoretical Grounding: Considerations for Hybrid Contexts

The parameters governing our model are grounded in established psychological research, but their instantiation is contextual. The shift to hybrid work invites specific hypotheses about how these foundational parameters might be systematically affected:

Gender and Susceptibility (sex): Empirical evidence suggests gender differences in emotional empathy and sensitivity. However, in hybrid environments reliant on digital communication, the reduction in non-verbal cues may attenuate these differences, as some channels of emotional expression are constrained. This suggests that the baseline susceptibility parameter associated with gender may require context-specific calibration for digital-heavy interactions.

Initial Emotional Intensity ($F{\left(i\right)}_{0}$): This parameter simulates early emotional disseminators. In hybrid work contexts, the threshold for becoming an effective “emotional seed” is likely higher, as initiating a contagion cycle through digital channels may require more explicit or intense expression to overcome the reduced social presence of digital media.

Cognitive Pathway Length (cognition): This determines the time required for reflective processing. As theorized in Section 3.3, the asynchronous nature of much hybrid communication may effectively lengthen the average cognitive pathway, providing more time for reflection before social pressure forces a stance. This parameter is key to modeling the potentially more deliberate identification processes in hybrid settings.

Attention (*d*): Operationalizing personal relevance, attention is paramount in hybrid work. In an environment saturated with digital notifications and competing cues, the ability of any single emotional stimulus to capture and sustain attention (*d*) is a critical bottleneck for memory accumulation and identity formation.

Enhancement Coefficient (rise%): This captures in-group reinforcement. The model allows us to hypothesize that within digital in-groups (e.g., tight-knit team channels), the rise% could be particularly high, simulating “echo chamber” effects that rapidly solidify sub-group identities.

These considerations do not invalidate our model’s general parameters but highlight its utility as a platform for generating and testing hybrid-work-specific hypotheses through targeted parameter variations. Table 2 is the specific parameters for the variables.

### 4.3. Model Dynamics and Mechanism Implementation

The core mechanisms of our theoretical framework are implemented through the following dynamic rules.

#### 4.3.1. Emotional Cycle Mechanism

An individual’s emotional susceptibility (*S*) is determined by their gender, attentional level, and the strength of their relationship with the influencing neighbor. The social relationship model introduces factors influencing conscious emotional contagion from a social interaction perspective, suggesting that women are more susceptible to emotional contagion than men. Based on this, this paper hypothesizes that women are more susceptible to emotional contagion from others than men. The variable *sex* is uniformly distributed between 0 and 1, where female =1 and male =0.

The attention factor *d* is introduced to measure an individual’s level of focus on emotional information conveyed by others. *a* represents an individual’s sensitivity factor to neighbors’ emotions, varying based on the hierarchical position of neighbors within their social network.

(1) $$S=b_{1}\times sex+b_{2}\times d+b_{3}\times a\left(j\right)\;a\left(j\right)=\left(j\in layer1:a_{1};j\in layer2:a_{2}\right)$$

The feedback factor (fb) represents social reinforcement from neighbors, intensifying the original emotional experience through social validation.

(2) fb=num(F(j)t>=susceptible)

#### 4.3.2. Emotional Memory Accumulation

The transient emotional experience triggered by stimulation gradually subsides and cools over time as the event recedes, though an individual’s sustained attention to the information can slow this decay. Let $F_{d1}\left(i\right)$ denote the emotional response decay value for each time step of a cell. decline1% represents the instantaneous emotional response decay rate at each time step. Equation (3) serves as the emotional experience decay rate function.

(3) $$Fd1{\left(i\right)}_{t}=F{\left(i\right)}_{t}\times decline1\%\times \left(1-d\right)$$

Long-term emotional memories formed from intense, homogeneous emotional experiences may also be forgotten, but their decay rate is significantly slower than that of immediate emotional responses. Let $F_{d2}\left(i\right)$ denote the decay value of an individual’s long-term emotional memory at each time step. decline2% represents the long-term emotional memory decay rate at each time step, where decline2%<decline1%. Equation (4) represents the long-term emotional memory forgetting rate function.

(4) Fd2(i)t=F(i)t×decline2%×(1−d)

Attention (*d*) moderates this decay, reflecting how personally relevant information maintains its emotional impact.

Equation (5) represents the emotional experience intensity function.

(5) $$F\left(i\right)t+1=\sum_{Moore}\frac{S\times F(j)t}{n}+a3\times F\left(i\right)t\times \left(1+fb\%\right)-Fd1\left(i\right)t$$

When the intensity of an individual’s emotional experience F(i) reaches infect%, it enters the process of emotional attitude formation and the mechanism of emotional memory accumulation.

#### 4.3.3. Cognitive Formation Mechanism

Equation (6) represents the emotional attitude formation function.

(6) $$F\left(i\right)t+1=\sum_{Moore}\frac{S\times F(j)t}{n}+a3\times F\left(i\right)t\times \left(1+fb\%\right)-Fd2\left(i\right)t+\alpha$$

The parameter α is a random value set to account for the influence of individual heterogeneity—such as social relationships and thought patterns—and the randomness of external social environment changes on emotional attitude tendencies, with Random∈[−1, 1].

Individuals with higher cognitive levels engage in longer and more complex processing before forming stable attitudes. The *cognition* parameter determines the duration of this reflective process, during which individuals integrate emotional experiences with personal values and organizational contexts. The tile attribute ‘*cognition*’ represents an individual’s cognitive level, while t=cognition denotes the length of the cognitive pathway formed. Higher ‘*cognition*’ values indicate greater cognitive capacity, resulting in longer periods of rational-dominated cognitive processing. When an S-state individual initially transitions to I-state, record the time point $t_{0}$ when they enter the cognitive formation stage. When the time step ticks reach $t_{0}+t$, observe the emotional attitude inclination of the I-state individual for the first time.

#### 4.3.4. Enhancement Mechanism

Equation (7) represents the affective attitude propensity function for ingroup members.

(7) $$F\left(i\right)t+1=\sum_{outgroup}\frac{S\times F(j)t}{n}+\sum_{ingroup}\frac{S\times (1+rise\%)\times F(j)t}{n}+a3\times F\left(i\right)t\times \left(1+fb\%\right)+\alpha$$

Due to the amplification effect mechanism, emotional contagion interactions among ingroup members reinforce emotional identification with the ingroup. This creates a positive feedback loop that solidifies group boundaries and strengthens identity.

The rise% metric measures the amplification level during emotional contagion interactions among ingroup members. Since stable emotional attitudes—specifically, attitudes aligned with the group’s emotional consensus—have already formed at this stage, these stable emotional attitudes do not fade over time. However, social mobility, competition, shifts in ingroup members’ social status or characteristics, changes in individual needs fulfillment levels, or the emergence of new identity objects can trigger deconstruction of identification with the original group. Therefore, a random fluctuation value α is incorporated into the affective attitude tendency function to account for this individual heterogeneity.

**Figure 4:**
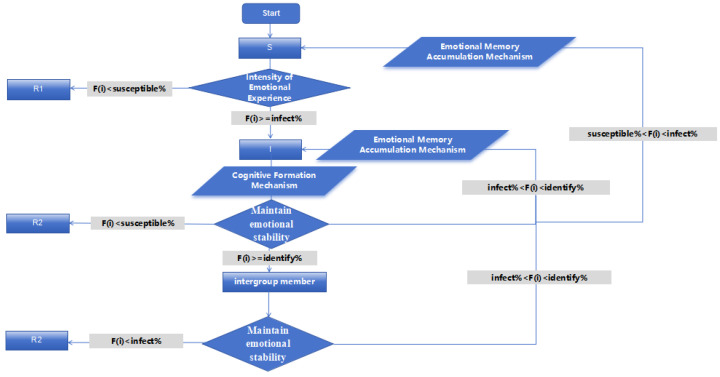
Flowchart of Cell State Dynamic Rules.

The specific process by which individual cells transition between the S, I, and R states is illustrated in Figure 4.

### 4.4. Model Validation, Robustness, and Pathways for Hybrid Work Application

**Figure 5:**
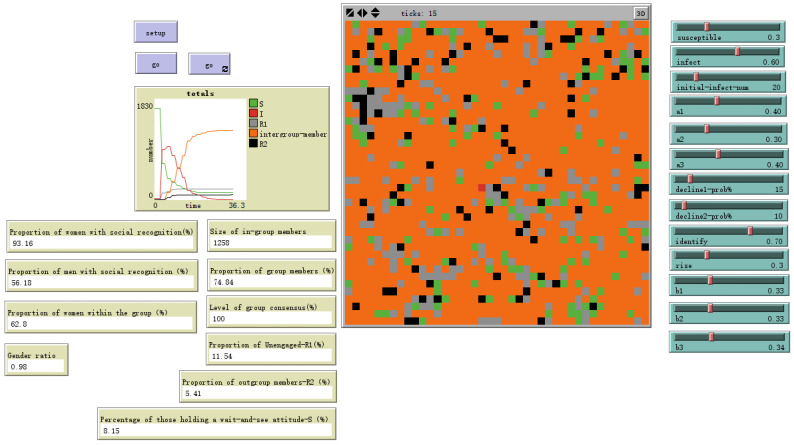
High attenuation rate-decline1 = 15%.

**Figure 6:**
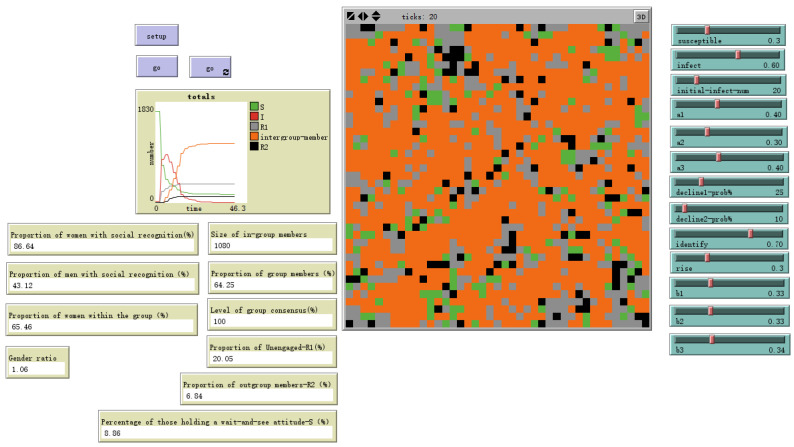
Low attenuation rate-decline1 = 25%.

To ensure the theoretical coherence and robustness of our model, we conducted systematic validation procedures. First, sensitivity analyses were performed to assess how variations in key parameters influenced the core outcomes of identity formation. For instance, we examined the impact of the immediate emotional decay rate (decline1%). As shown in Figure 5 and Figure 6, when this parameter was varied across a plausible range (15% to 25%), the resulting proportion of agents forming a stable in-group identity fluctuated within a bounded range of 65% to 74%. This indicates that the model is meaningfully responsive to this theoretically grounded parameter—faster decay reduces the pool of sustained emotional arousal available for contagion—while ultimately producing robust and consistent qualitative outcomes (i.e., a clear in-group/out-group segmentation) across its range.

**Figure 7:**
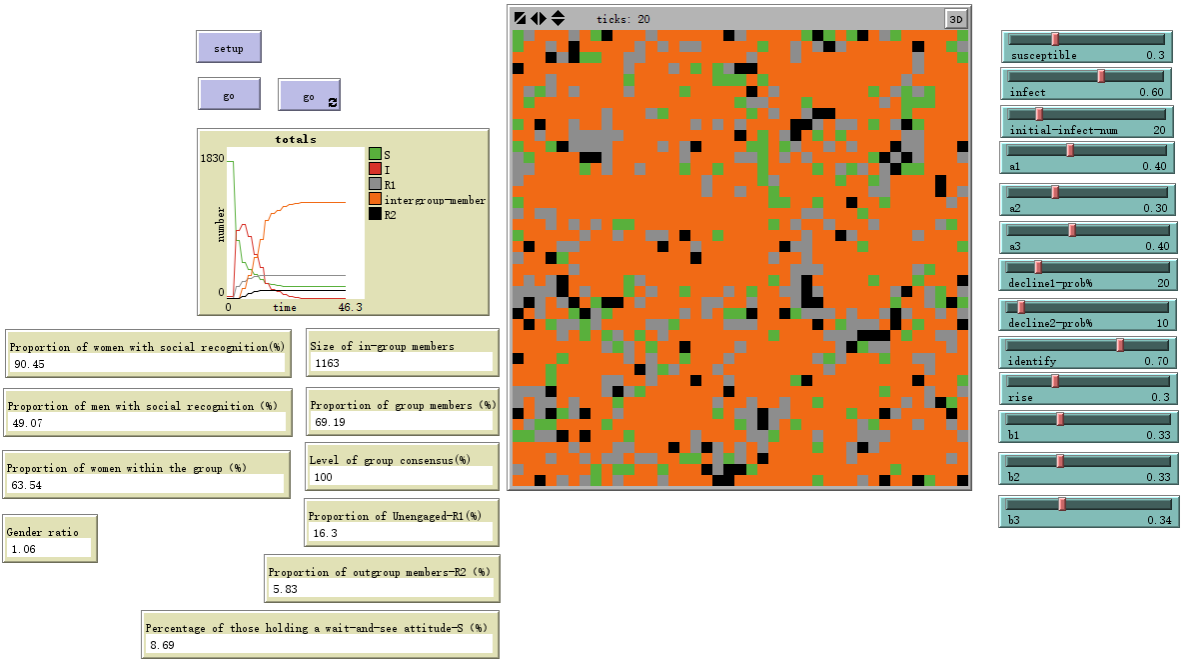
High reinforcement rate-rise = 30%.

**Figure 8:**
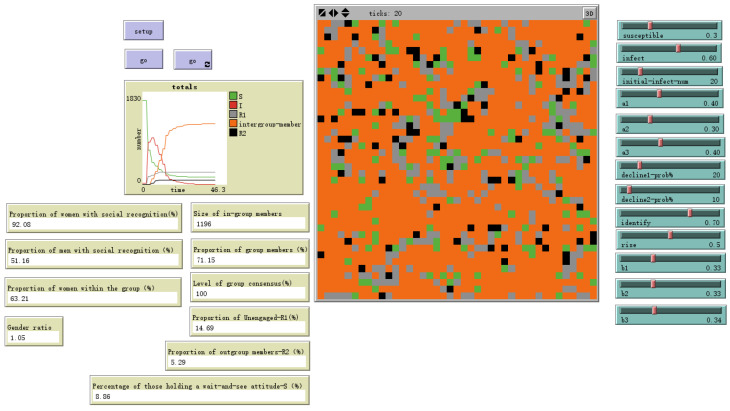
Low reinforcement rate-rise = 50%.

Similarly, the model demonstrated expected sensitivity to the enhancement coefficient (rise%), as shown in Figure 7 and Figure 8, where higher values accelerated in-group consensus formation but also increased the risk of stark polarization. These analyses confirm that the model behaves in a theoretically predictable and stable manner.

**Figure 9:**
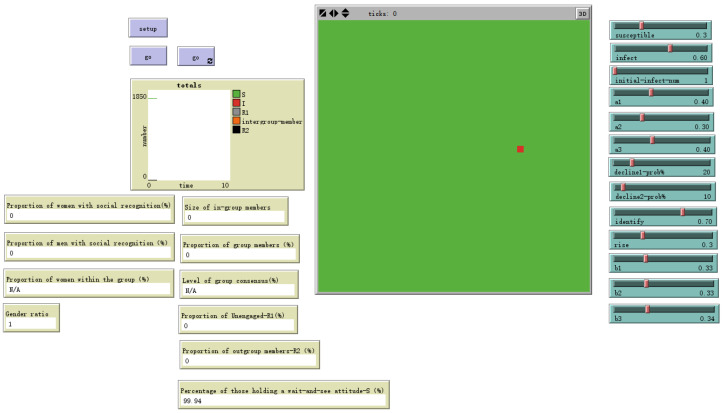
Number of initial spreaders = 1.

**Figure 10:**
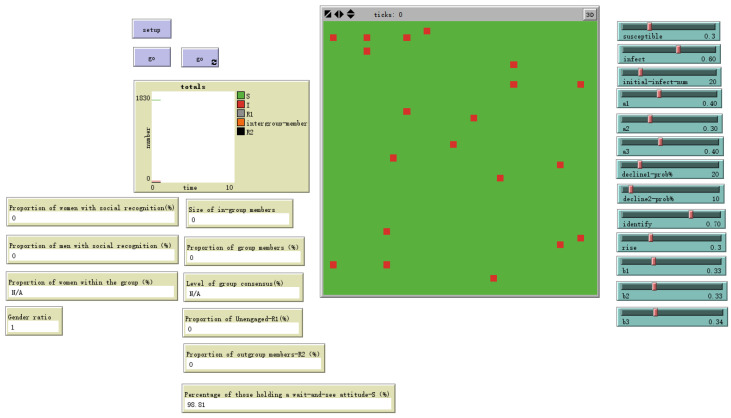
Number of initial spreaders = 20.

Second, we assessed the model’s robustness to different initialization conditions. Beyond the randomized seeding of initial “infected” agents used in our primary analyses, we tested alternative configurations, including seeding from a single central agent or from multiple clustered seeds, as shown in Figure 9 and Figure 10. While the specific spatial pattern of early identity formation varied, the final macro-level outcomes—specifically, the equilibrium distribution of in-group, out-group, and unengaged agents—remained consistent across these different initial conditions. This suggests that the emergent identity landscape is a product of the defined interaction mechanisms and network structure, rather than an artifact of a particular starting setup.

Finally, the model’s emergent patterns were evaluated for face and construct validity. The simulation reliably generated phenomena well-documented in organizational life, such as the formation of cohesive subcultures (in-groups), the persistence of skeptical minorities (out-groups), and the presence of disengaged individuals. These patterns, emerging from the bottom-up interactions of simple agents, align with complex organizational realities, thereby supporting the model’s construct validity.

A critical strength of this computational approach is its capacity for future empirical calibration and testing, particularly within hybrid work contexts. It is important to acknowledge that the parameter values used in the present simulations are theoretically derived, serving to instantiate and test our proposed mechanisms. The next essential step is the contextual calibration of these parameters using empirical data from hybrid organizations. This would involve harvesting data such as digital communication patterns (e.g., frequency and sentiment of emails/chat messages), longitudinal survey measures of organizational identification and perceived emotional culture, and digital trace data mapping actual interaction networks and their strength.

Calibrating model parameters (e.g., baseline susceptibility, emotional decay rates, the enhancement coefficient rise%) against such real-world datasets would transform the model from a purely theoretical tool into a quantitatively calibrated representation of specific organizational contexts. Subsequently, running simulations under these empirically informed parameter configurations would generate testable, nuanced predictions about identity formation trajectories in hybrid settings. Comparing these model-derived predictions with observed longitudinal outcomes in partner organizations would constitute a powerful form of empirical validation. This approach positions our model not as a closed system with fixed parameters, but as a generative and adaptable theoretical platform. It provides a clear methodological pathway for future research to engage directly with the empirical realities of hybrid work, thereby bridging computational theorizing with rigorous empirical testing and offering a dynamic complement to traditional methods in organizational psychology.

## 5. Results

The simulations generated rich data on the emergent outcomes of the emotional contagion and identity formation processes. We present the results not merely as model outputs, but as evidence illuminating the dynamic interplay between emotion, cognition, and social structure in organizational identity formation. Table 3 presents the variable settings for the model under each scenario described below.

### 5.1. The Emergent Landscape of Organizational Identity

Under the baseline configuration, the model reached a stable equilibrium, revealing a clear segmentation of the organizational population consistent with theoretical expectations. The results demonstrate that the process of emotional contagion does not lead to uniform assimilation but rather to a distinct crystallization of group affiliations.

**Figure 11:**
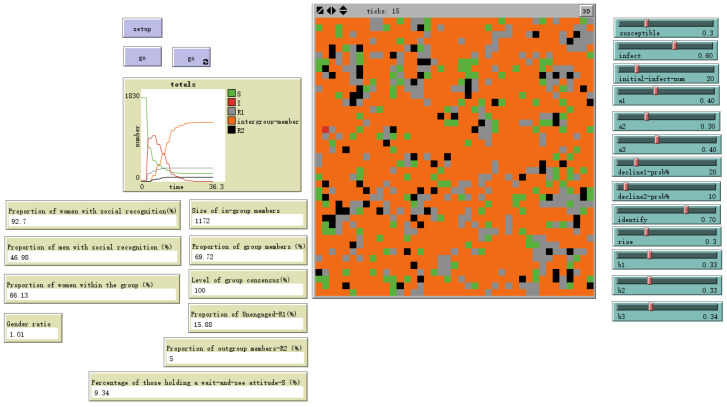
Model Evolution Results Under Basic Settings with male sex value = 0 and female sex value = 1.

As shown in Figure 11, the final population comprised four psychologically meaningful groups:

- **In-group (69.72%)**: Individuals who develop stable social identification aligned with emerging group consensus through emotional contagion and cognitive formation processes, i.e., I-state individuals (orange tiles).
- **Out-group (5%)**: Individuals who actively reject and maintain non-alignment with the group’s emotional consensus after cognitive processing, thereby achieving cognitive immunity, i.e., R2-state individuals (black tiles).
- **Non-participants (15.88%)**: Individuals whose emotional arousal subsided before triggering sustained cognitive processing, reverting to a neutral, non-engaged state—R1-state individuals (gray tiles).
- **Observers (9.34%)**: Individuals who exhibit some emotional response to the stimulus but have not yet formed a stable attitudinal orientation, i.e., S-state individuals (green tiles). Unlike non-participants—who achieve emotional immunity through immediate emotional decay and subsequently cease emotional contagion—observers remain susceptible to ongoing emotional contagion.

This distribution validates the model’s core premise: a pathway exists from emotional arousal to social identity, but it is moderated by individual-level characteristics that determine whether an individual completes the journey. In hybrid work contexts, this segmentation suggests that fostering a unified organizational identity may require targeted strategies to engage the “Unengaged” and bridge the gap with the “Out-group,” rather than assuming homogeneous assimilation.

The above results represent the evolutionary outcomes when female sex value = 1 and male sex value = 0. To simulate gender effects in different organizational contexts and validate the impact of gender differences on outcomes, a sensitivity analysis is conducted below.

**Figure 12:**
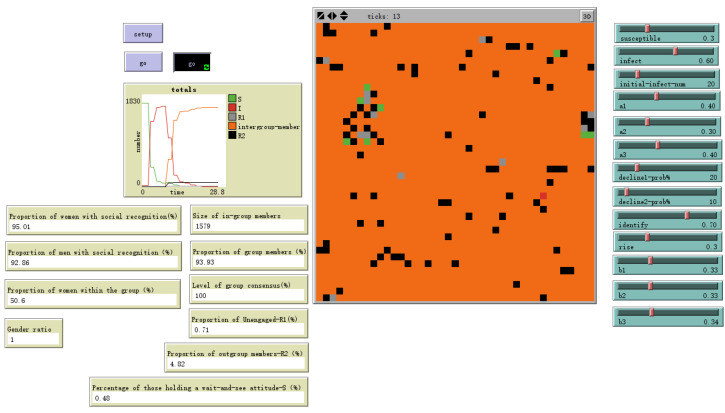
Evolutionary outcome with male sex value = 0.5 and female sex value = 1.

As shown in Figure 12, when gender differences in susceptibility to emotional information are smaller and individual emotional susceptibility is higher, setting male sex value = 0.5 and female sex value = 1 yields the evolutionary results shown in the figure. The gender ratio disparity among ingroup members narrows, with females outnumbering males by 1.2%. Simultaneously, the overall ingroup proportion increases by 24.21 percentage points.

**Figure 13:**
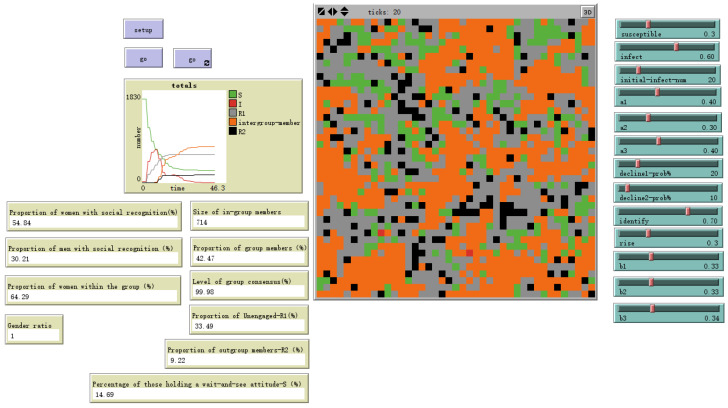
Evolutionary results with male sex value = 0 and female sex value = 0.5.

As shown in Figure 13, when the susceptibility to emotional information differs little between genders and individual emotional susceptibility is low, setting male sex value = 0 and female sex value = 0.5 yields the evolutionary outcome shown. The gender ratio disparity among ingroup members narrows, with females outnumbering males by 28.58%. At this point, the overall ingroup proportion decreases to 42.47%.

To better observe how different genders exhibit varying responses to other factors influencing the formation and reinforcement of group identity, thereby developing organizational communication strategies that account for gender differences, variable settings that more prominently highlight behavioral differences between men and women can achieve better model performance. Therefore, the following settings are adopted: femalesex=1, malesex=0.

### 5.2. The Critical Moderating Role of Cognitive Level

**Figure 14:**
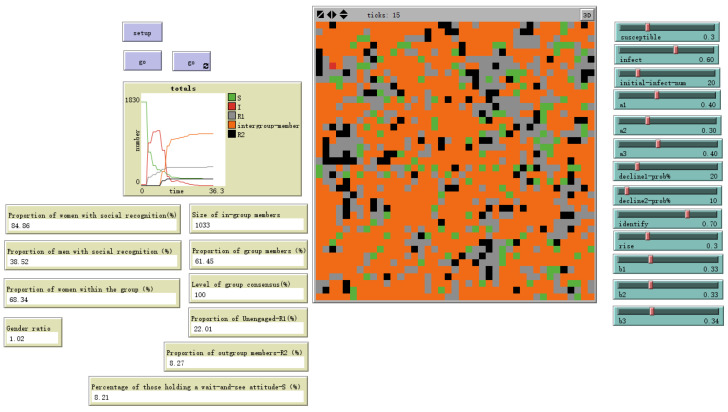
Evolutionary Results of Models with Higher Cognitive Levels-Cognition Random (3, 4, 5).

**Figure 15:**
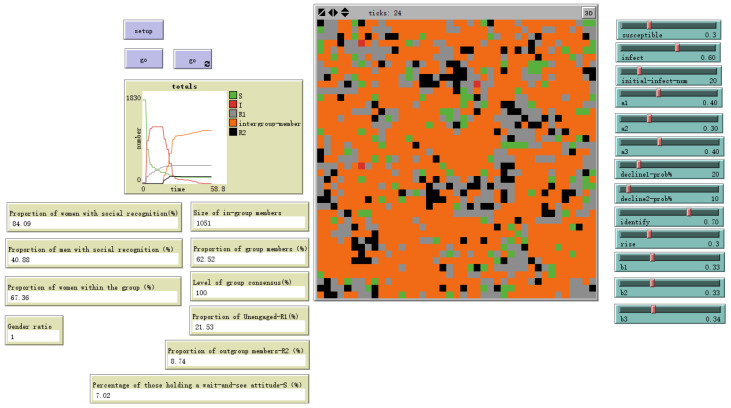
Evolutionary Results of Models with Higher Cognitive Levels-Cognition Random (6–10).

A key finding concerns the impact of cognitive heterogeneity. When we simulated an organization with a higher average cognitive level (setting cognition to 3, 4, or 5), the proportion of individuals forming a social identity decreased to 61.45%, a reduction of 8.27 percentage points from the baseline. When cognitive settings were elevated to higher levels (6–10), the proportion of individuals forming identification further decreased to 62.52%, as shown in Figure 14 and Figure 15.

This result underscores a critical nuance: higher cognition acts as a buffer against rapid assimilation. Individuals with greater cognitive resources undergo longer and more complex evaluation processes, thereby reducing the likelihood of uncritically internalizing mainstream emotional consensus.

### 5.3. The Impact of Emotional and Attentional Factors

#### 5.3.1. Initial Emotional Arousal

**Figure 16:**
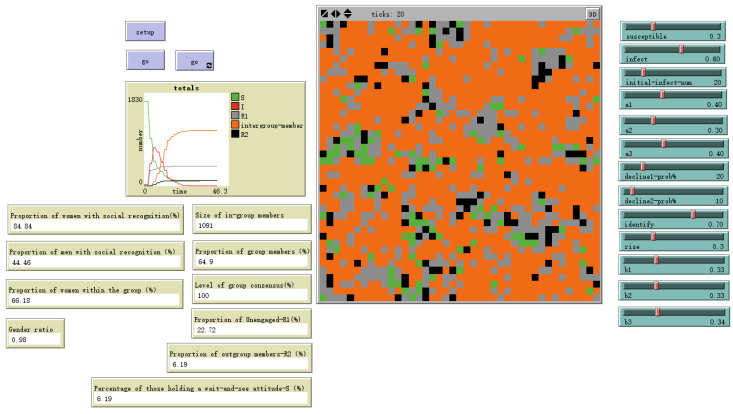
Initial Emotional Arousal Intensity is Low-S State Cell at time = t0, F(i) Random (0.3, 0.4).

The intensity of initial emotional arousal has been demonstrated to be a powerful catalyst. To simulate model evolution outcomes when group initial arousal intensity is low, the initial state of S-state cells was set to randomly draw continuous values within [0.3, 0.4] for emotional experience intensity. This was compared against results from the baseline setting where S-state cells initially exhibited emotional experience intensity within [0.3, 0.6]. As shown in Figure 16, by time step 19, the proportion of individuals forming social identification reached 64.9%, a decrease of approximately 4.82% compared to the 69.72% observed under the baseline setting. The proportion of non-participating individuals reached 22.72%, which is 1.43 times that of the baseline setting. This demonstrates that the initial emotional arousal intensity significantly influences emotional contagion interactions. When the group’s initial emotional arousal intensity is low, the emotional experience of a large proportion of individuals rapidly diminishes over time due to its decay. This leads to emotional immunity, where individuals transition from the S state to the R1 state. Once emotionally aroused, they cease to engage in sustained emotional transmission and sharing.

This indicates that without sufficient emotional “fuel” to meet the threshold, the emotional feedback loop cannot sustain itself. A significant portion of the population (22.72%) experienced emotional arousal that faded before influencing cognition or driving transmission, leading to their disengagement.

#### 5.3.2. The Role of Attention and Personal Relevance

**Figure 17:**
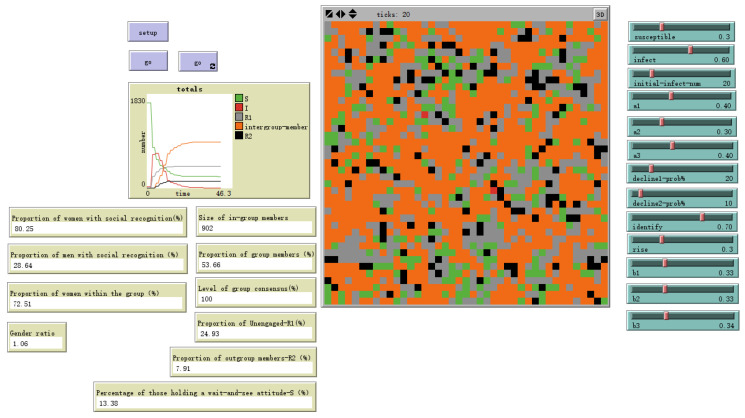
Low Attention—*d* Random (0–0.8).

Operationalizing the personal relevance of issues as the attention factor is one of the most influential elements in this model. To simulate situations where groups pay less attention to emotional information, the individual attention factor was set to randomly take continuous values within [0, 0.8], and the results were compared with those under the baseline setting where the attention factor for S-state individuals ranged from [0, 1]. As shown in Figure 17, by time step 20, the proportion of individuals forming social conformity reached 53.66%, a decrease of approximately 16.06% compared to the baseline setting’s 69.72%. Among males, the proportion forming social conformity was 28.64% of the total male population, a decrease of about 18.34% from the baseline setting’s 46.98%. In contrast, the proportion of females forming social conformity decreased by only about 12.45%. Under the low attention factor, the proportion of females within ingroups increased from 66.13% in the baseline setting to 72.51%. This further indicates that females, being more susceptible to emotional contagion, exhibit a higher probability of forming identification with groups less relevant to themselves.

**Figure 18:**
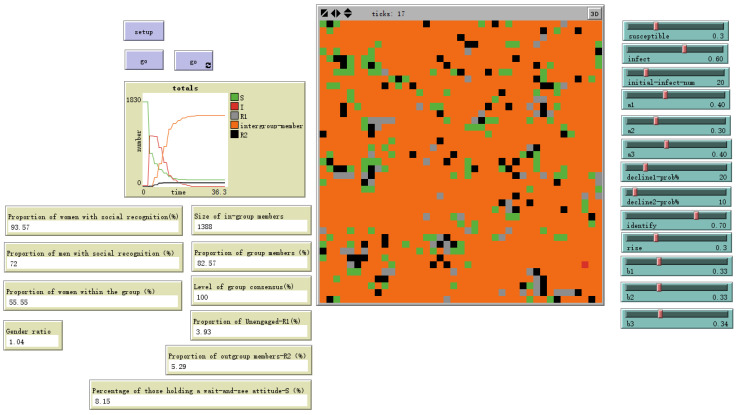
Low Attention—*d* Random (0.2–1).

As shown in Figure 18, when the attention factor *d* is set to randomly continuous values within [0.2, 1], the proportion of in-group members forming social identification increases by approximately 12.85% compared to when *d* is randomly sampled within [0, 1]. The proportion of social identification among females increased by only 0.87%, while that among males rose by 25.02%. This indicates that males’ probability of forming social identification is significantly influenced by the closeness of the connection between the emotionally charged information and their own interests, or their level of interest. Males are less likely to develop emotional resonance with groups that do not directly concern them.

This indicates that male identification is highly contingent on perceived personal relevance, whereas female identification, while also benefiting from relevance, is less dependent on it due to a generally higher baseline susceptibility to emotional contagion.

### 5.4. The Dynamics of Consensus Formation and Its Hybrid Work Implications

Finally, the model provided insight into the consolidation phase of identity. Once formed, the in-group exhibited a very high “Group Consensus Degree” (100% in the baseline scenario), reflecting strong homogeneity in affective attitudes. This outcome is driven by the Enhancement Effect mechanism, where interactions within the in-group are amplified, creating a positive feedback loop that solidifies the shared identity and insulates it from alternative viewpoints. This illustrates how organizational subcultures can become highly cohesive and resistant to change once a critical mass of identified members is established. This mechanism has potent implications for hybrid work, where digital in-groups (e.g., team channels) can easily become high-fidelity “echo chambers.” The model suggests that without deliberate intervention to foster cross-group exposure, hybrid organizations may experience accelerated silo formation and entrenched subcultures.

The preceding results demonstrate that our model successfully replicates the core dynamics of identity formation and consolidation as theorized. A pivotal insight from this validation is that the strength of the Enhancement Effect, and indeed the efficiency of the entire identity formation pathway, is intrinsically linked to the underlying structure and interaction patterns of the organization. This leads to a critical, applied question: how do these pathways and outcomes systematically differ when the fundamental organizational fabric shifts from a traditional, co-located setting to a highly dispersed hybrid one? To answer this question and directly leverage the model’s capability for comparative analysis, we conducted a dedicated set of exploratory simulations contrasting high- and low-hybridity configurations.

### 5.5. Exploratory Simulation: Contrasting High- vs. Low-Hybridity Configurations

Building directly on the understanding that identity dynamics are context-dependent, we conducted a set of exploratory simulations to interrogate how hybrid work reconfigures these pathways. Specifically, we contrast two archetypal organizational configurations: a Low-Hybridity setting, which approximates a traditional, co-location-centric workplace, and a High-Hybridity setting, representing a highly dispersed, digitally dependent organization. This comparative approach shifts our model from a general theoretical framework to a specific diagnostic instrument for understanding hybrid work dynamics.

Simulation Design: We operationalized these configurations by manipulating key parameters linked to the theoretical mechanisms and the structural features of hybrid work (see Table 4 for a summary).

Low-Hybridity Configuration: Reflecting high-frequency, rich, in-person interaction, we increased the influence strength of the Primary Network (simulating strong, immediate collegial ties) and set a higher feedback factor (fb) to model the potent, immediate social reinforcement typical of face-to-face exchanges.

High-Hybridity Configuration: Mirroring a workforce with minimal co-location, we reduced the density and tie strength of the Primary Network (simulating fewer and weaker strong ties due to physical separation). Concurrently, we lowered the feedback factor (fb) to capture the attenuated and delayed emotional feedback characteristic of digital communication (e.g., text, asynchronous video).

**Figure 19:**
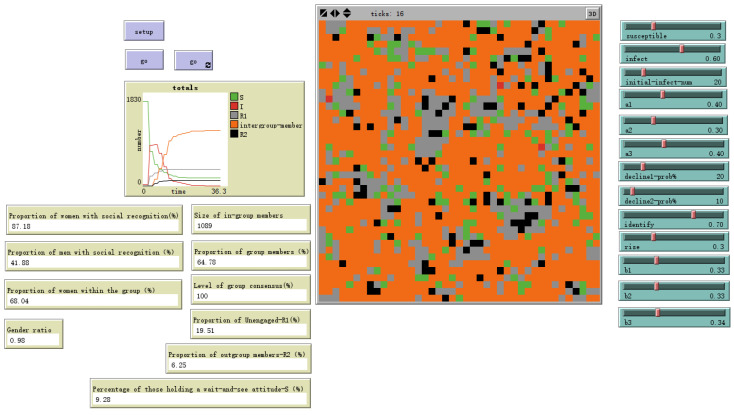
Evolutionary Results at High Mixing Degree—Feedback Factor fb Reduced by 50%.

As shown in Figure 19, the results from these contrasting simulations revealed markedly divergent trajectories of identity formation, providing a quantitative illustration of the challenges inherent to high-hybridity work. A primary finding concerned the speed of identity development. The model reached a stable equilibrium significantly faster under the Low-Hybridity configuration. In the High-Hybridity setting, the time required to achieve a stable distribution of identities increased by approximately 3 time steps. This substantial delay can be directly attributed to a weakening of the Emotional Cycle mechanism. The lower feedback factor (fb) means each emotional exchange generates less social amplification, requiring more interaction cycles to build the affective momentum necessary for widespread contagion.

Furthermore, the scale and composition of the resulting in-group differed meaningfully between configurations. The final proportion of agents forming a stable in-group identity was roughly 5% smaller in the High-Hybridity simulation. This outcome stems from the interplay of two mechanisms. First, the less efficient Emotional Cycle failed to propagate emotions to some peripheral agents before their initial emotional arousal decayed. Second, and perhaps more critically, the Cognitive Formation mechanism assumed a more prominent filtering role. In an environment characterized by weaker direct social pressure (simulated by lower Primary Network strength), individuals—particularly those with higher inherent cognitive reflection (cognition values)—demonstrated a greater propensity to cognitively appraise and, in some cases, reject the emerging group consensus rather than assimilate uncritically. Consequently, the resulting in-group, while potentially more robust due to this deliberate reflection, was numerically smaller.

Finally, the nature of the consensus that emerged also varied. While both configurations ultimately fostered a high degree of in-group affective homogeneity, the pathways to this consensus were distinct. In the Low-Hybridity setting, consensus emerged broadly and uniformly across the network. Under High-Hybridity conditions, consensus formed more rapidly within isolated digital sub-clusters—simulated by the remaining pockets of strong Primary Network ties—where the Enhancement Effect (rise%) acted powerfully within these digital “echo chambers” to rapidly solidify local identities. Achieving organization-wide consensus then depended on the slower, less efficient diffusion processes across the weaker Secondary Network, highlighting a tangible risk of persistent silos and fragmented subcultures without deliberate integrative intervention.

In summary, this exploratory contrast renders the preceding theoretical arguments concrete and measurable. It demonstrates that the shift to hybrid work transcends a mere change in location; it constitutes a fundamental alteration of the social-psychological engine that drives organizational culture. High-hybridity configurations, by structurally dampening the Emotional Cycle and reshaping interaction networks, predispose organizations toward identity formation processes that are slower, more susceptible to fragmentation, and potentially more exclusive. These insights provide a quantified, dynamic basis for the managerial challenges and strategic interventions discussed in Section 6.2.1, underscoring the value of our model as a platform for anticipating the cultural implications of specific hybrid work policies.

## 6. Discussion

The present study set out to illuminate the “black box” linking momentary emotional experiences to the development of enduring social identities in organizations. By building and simulating a computational model grounded in organizational theory, we have moved beyond static correlations to expose the dynamic, multi-stage process through which macro-level culture emerges from micro-level affective interactions. Our findings offer nuanced theoretical insights, actionable managerial implications, and a clear agenda for future research, with particular resonance for the evolving nature of work. Compared to existing research in organizational identity, this study offers a mechanistic explanation of how micro-level emotional interactions promote macro-level cultural stability. Building upon this foundation, we highlight the moderating roles of cognitive heterogeneity, attentional focus, and gender differences. Furthermore, we conceptualize identity construction as a diachronic process influenced by accumulated emotional memories, introducing a temporal depth dimension. This framework offers new insights into how organizations in hybrid contexts can consciously design emotional interaction scenarios and accumulate positive collective memories to mitigate the challenges of weakened identity arising from spatiotemporal separation.

### 6.1. Theoretical Contributions

Organizational identity is a dynamic, emergent property of complex social psychological systems. It originates from shared emotional resonance, takes shape through the gradual accumulation of memories and deliberate cognitive processes, and is continuously reinforced by the strengthening of group boundaries. Our primary contribution lies in formalizing and testing a cross-level dynamic framework that explains how transient affective pulses crystallize into stable identity structures. This framework bridges a critical chasm in organizational psychology between the micro-foundations of emotion and the macro-phenomena of culture and identity.

First, our “Four-Stage, Four-Mechanism” model provides a mechanistic explanation for a process often described in metaphorical terms. We demonstrate that identity is not a static trait but a dynamic outcome of a system driven by emotional cycles, memory accumulation, cognitive appraisal, and enhancement. This advances theories of organizational culture (Hatch & Schultz, 2002) by specifying precisely how members’ immediate emotional reactions to events—a successful launch, a restructuring announcement—can, through repeated social interactions, sediment into enduring commitment to, or alienation from, the organization.

Second, the results clarify the critical yet complex moderating role of cognitive heterogeneity. We found that a higher group cognitive level did not prevent identity formation but regulated its speed and robustness. The slower, more deliberate identification pathway of high-cognition individuals offers a fresh perspective for understanding the “silent minority” or “critical thinkers” within organizations. Individuals with greater cognitive resources underwent a longer and more complex appraisal process, reducing the likelihood of uncritically internalizing the prevailing emotional consensus. Consequently, identity formation was slower and the final in-group was smaller but potentially more robust, as membership was based on more deliberate reflection. This finding helps explain the presence of “thoughtful silos” or “critical minorities” within organizations, whose delayed or conditional buy-in should not be misinterpreted as disloyalty but as a different pathway to identification. This finding underscores the particular importance of accommodating longer cognitive processing times in hybrid settings, where asynchronous communication may already naturally extend reflection periods and where pressuring for rapid consensus could be counterproductive. This finding resonates with conceptualization of cognitive diversity as a resource for team innovation, while also highlighting the temporal dimension of identity formation that has been underemphasized in prior work (van Knippenberg et al., 2020). Their delayed alignment may not signify a lack of loyalty but rather a more protracted and effortful cognitive process.

Third, by incorporating the emotional memory accumulation mechanism, our model conceptualizes identity formation as a diachronic process. This underscores that building a strong organizational identity cannot be achieved through a single emotional event; it relies on the cumulative impact of multiple affective experiences that collectively weave the organization’s “affective memory fabric.” This directly positions organizational narratives, rituals, and symbols—the key carriers of emotional memory—at the heart of cultural construction and maintenance.

### 6.2. Practical Insights

#### 6.2.1. Management Insights: From Emotional Management to Cultural Development

Our findings provide organizational leaders with a strategic roadmap for moving from passively managing emotional outbursts to actively and intelligently shaping a robust organizational identity.

Strategically Designing Affective Events: Managers should identify and deliberately design “key moments” that can initiate positive emotional cycles (e.g., project celebrations, public recognition). Conversely, for negative events, immediate and transparent intervention is crucial to disrupt the cyclical amplification of negative affect and guide the cognitive appraisal process toward constructive sense-making.

Fostering a Culture of Cognitive Inclusion: Organizations must avoid misinterpreting thoughtful silence for disloyalty. Creating an environment that permits deep reflection and rational debate is essential for fostering the higher-quality, more resilient identification that emerges from deliberate cognition. This may require adapting leadership communication and performance systems to reward constructive critical thinking.

Leveraging the Enhancement Effect: Once a positive group consensus emerges, managers can consciously exploit the enhancement mechanism. This involves strengthening in-group markers (e.g., unique value propositions, team rituals), framing shared external challenges, and curating in-group success stories to continuously reinforce the boundaries of “who we are.”

Tailoring Communication Strategies: The confirmed gender-based differences in susceptibility and reliance on personal relevance suggest practical considerations. When building teams where rapid cohesion is paramount, gender composition can be a factor. Furthermore, communications aimed at fostering identification among male members should more explicitly articulate the relevance to personal interests and overarching goals. Communication strategies aimed at fostering organizational identity may require adjustments, with messages to male employees needing to more clearly articulate their direct relevance to their roles and interests. In a dispersed hybrid environment often saturated with digital noise, capturing and sustaining attention (d) presents a significant challenge. These findings indicate that organizations must exercise extreme caution in constructing messages to convey signals of relevance, particularly to effectively engage male employees in identity-building processes.

Enhancing Initial Emotional Arousal Intensity: For organizational initiatives to spark identity resonance, a baseline level of genuine emotional engagement must first be generated. In hybrid work settings, this implies that digital or hybrid communications aimed at identity building may need to be designed with greater emotional vividness and salience to overcome the “colder” nature of the medium and reach the required communication threshold.

#### 6.2.2. Implications for Understanding and Improving Hybrid Work Environments

Our model’s dynamic, mechanism-based perspective offers unique value for diagnosing and addressing the core cultural challenges of hybrid work. It reframes key hurdles as disruptions to specific stages of the identity–formation pathway, thereby revealing targeted intervention points.

Understanding the Challenges: The model helps explain why hybrid work can struggle with cultural cohesion: (1) Emotional Fuel Shortage: Attenuated digital feedback weakens the Emotional Cycle, making it harder to achieve the critical mass of shared affect needed to kickstart identity formation. (2) Fragmented Memory Formation: The reduction in shared, high-intensity co-located events challenges the Emotional Memory Accumulation mechanism, risking a thin or fractured affective bedrock for culture. (3) Asynchronous Cognitive Drift: While providing reflection time, elongated cognitive pathways may delay consensus and allow interpretations to diverge without the synchronizing pressure of physical presence.

Actionable Levers for Hybrid Work Management: Based on our mechanisms, we propose concrete practices:

Designing Hybrid Affective Rituals: Consciously engineer “hybrid moments” for key emotional stimuli. Ensure remote participants have equally intense, interactive, and perceptible channels (e.g., structured virtual breakout rooms during an all-hands celebration, synchronized digital celebrations) to fuel the Emotional Cycle equitably.

Managing Digital Emotional Cues: Train teams in digital emotional literacy—explicitly articulating sentiment in text, using video to convey non-verbal cues, and norming responsive feedback (even simple reactions). This maintains the feedback loop (fb) critical for contagion in digital mediums.

Building Digital Identity Anchors: Proactively create and curate digital repositories of shared symbols, milestone achievements, and collective stories. These become the new, accessible carriers of Emotional Memory and focal points for the Enhancement Effect, forging a common digital “homeroom” for dispersed employees.

Harnessing Asynchronous Reflection: Formalize the use of asynchronous platforms (e.g., internal forums, shared documents) for complex discussions. This respects the extended Cognitive Formation pathway (cognition) of individuals, leveraging the hybrid environment’s native strength to foster deeper, more considered buy-in rather than pressuring instant, superficial consensus.

Our computational framework serves as a vital tool for hybrid work research. It allows scholars to act as “policy simulators,” testing the long-term cultural implications of different hybrid configurations in silico before costly real-world implementation. For example, researchers can systematically vary parameters to model: the impact of mandated versus flexible office days on network structure and consensus speed; the effect of different primary communication tools (e.g., video-first vs. text-first) on emotional contagion efficiency; or how different sub-group clustering algorithms in digital platforms influence silo formation via the Enhancement Effect (rise%).

A crucial theoretical implication of our model is its inherent suitability for conceptualizing hybridity not as a binary state (remote vs. office), but as a continuous variable. Hybrid work exists on a spectrum defined by the proportion of time spent co-located, the blend of communication media, and the resulting structure of social networks. Our model’s parameters—such as the relative density and tie strength of the Primary versus Secondary networks, the feedback factor (fb), and the attention parameter (*d*)—are naturally suited to operationalize this continuum. By systematically varying these parameters, future research can move beyond comparing discrete “hybrid” and “non-hybrid” conditions to map the non-linear relationships between degrees of hybridity (e.g., varying levels of in-person work frequency) and the dynamics of identity formation. This positions our framework to explore nuanced questions, such as whether there are critical thresholds of physical co-presence necessary to sustain certain identity-forming mechanisms, or how different dimensions of hybridity (temporal, spatial, technological) interact. This capability significantly expands the model’s theoretical utility, transforming it into a generalizable platform for understanding organizational identification across the full spectrum of modern work arrangements.

For organizations navigating the complexities of hybrid work, understanding and leveraging these micro-dynamics through the lens of our model is not an academic exercise but a strategic imperative. It provides a evidence-based framework for intentionally cultivating a cohesive, adaptive, and resilient culture, regardless of where work takes place.

### 6.3. Limitations and Future Research

While our model provides a powerful theoretical tool, its generalizability must be considered. The model’s parameters are likely context-dependent. In real-world organizations, interpersonal relationships are more complex, and connections between individuals often exhibit asymmetry. That is, in emotional information exchange, the impact of emotional contagion varies between parties due to differences in the direction of emotional information transmission. Furthermore, in hybrid work settings, novel collaborative approaches facilitated by digital communication platforms enable individuals to establish connections across broader and more diverse networks. This study, however, simplifies neighbors into two categories: those with closer relationships and those with more distant relationships. Consequently, the model carries inherent risks of failure when attempting to simulate the complex scenarios described above.

In terms of technical limitations, the research model presented in this paper is based on theoretical deduction and lacks validation and calibration using real organizational data. Future research should empirically calibrate these parameters using real-world data, such as sentiment analysis of organizational communication.

Furthermore, our model opens up several avenues for future inquiry: Cross-Cultural Comparisons, investigating dynamics in different Organizational Typologies, and Incorporating Power and Hierarchy to simulate how power asymmetries shape emotional contagion.

## Figures and Tables

**Table 1:** Cell State Types.

F(i)		State		
F(i)<susceptible%		Immune individuals		-
susceptible%≤F(i)<infect%		Susceptible individuals		Emotional
F(i)≥infect%	F	Infected individuals	Ordinary	Identity
	F(i)<identify%		In-group	Identity

**Table 2:** Model Variable Settings.

Cell Attribute	Variable	Value	
Sex	Sex	Random (0, 1)	
Attention	*d*	Random ∈ (0, 1)	
Initial Emotional Intensity	$F{\left(i\right)}_{t_{0}}$	I-State	Random ∈ [0.6, 0.7]
		S-State	Random ∈ [0.3, 0.6]
Cognitive Pathway Length	Cognition	Random (1, 2, 3, 4, 5)	
Random Fluctuation Value	α	Random ∈ [−1, 1]	
Emotional Decay Rate	decline1%	20%	
Emotional Decay Rate	decline2%	10%	
Enhancement Coefficient	rise%	30%	

**Table 3:** Simulation Variable Settings.

Simulated Scenario	Variable Settings
the susceptibility to emotional information differs little between genders and individual emotional susceptibility is low	male sex value = 0, female sex value = 0.5
the susceptibility to emotional information differs little between genders and individual emotional susceptibility is high	male sex value = 0.5, female sex value = 1
Evolutionary Results of Models with Higher Cognitive Levels	Cognition Random (3, 4, 5)
	Cognition Random (6–10)
Initial Emotional Arousal Intensity is Low	S State Cell at time = $t_{0},\;F\left(i\right)$ Random (0.3, 0.4)
Low Attention	*d* Random (0, 0.8)
High Attention	*d* Random (0.2, 1)
High-Hybridity (Dispersed Hybrid)	F(b) Reduced by 50%

**Table 4:** Parameter Configurations for Exploratory Simulations.

Parameter	Low-Hybridity (Traditional Office)	High-Hybridity (Dispersed Hybrid)	Theoretical Rationale
Primary Network Strength	High	Low	Simulates the frequency and intensity of core team interactions.
Feedback Factor (fb)	Consistent with the basic settings	Reduced by 50%	Represents the efficiency of the emotional feedback loop.
**Illustrative Outcome Metrics**			
Time to Stable Consensus	ticks = 13	ticks = 16	Speed of identity formation.
Final In-Group Size	69.72%	64.78%	Proportion achieving stable identification.
Group Consensus Degree	Very High	High (but potentially more fragmented)	Homogeneity of affective attitudes within the in-group.

## Data Availability

This study is based on computational modeling and does not involve the collection of empirical human data. All simulations were performed using an agent-based model implemented in NetLogo. The model code, parameter settings, and simulation outputs are available upon request from the corresponding author.
